# The Effect of Calcitonin Gene-Related Peptide Monoclonal Antibodies on Quality of Life among Migraine Patients: Pilot Study at the Hospital of Lithuanian University of Health Sciences Kaunas Clinics

**DOI:** 10.15388/Amed.2024.31.1.12

**Published:** 2024-02-27

**Authors:** Monika Remenčiūtė, Greta Varžaitytė, Gintarė Žemgulytė

**Affiliations:** 1 Kaunas Hospital of the Lithuanian University of Health Sciences, Kaunas, Lithuania; 2 Lithuanian University of Health Sciences, Medical Academy, Faculty of Medicine, Department of Neurology, Kaunas, Lithuania

**Keywords:** chronic migraine, calcitonin gene-related peptide monoclonal antibodies, Migraine Disability Assessment, 36-Item Short Form Survey Instrument questionnaire, lėtinė migrena, su kalcitonino genu susijusį baltymą ir jo receptorius veikiantys monokloniniai antikūnai, migrenos įtakos veiklai (MIDAS) klausimynas, su sveikata susijusios gyvenimo kokybės (SF-36) klausimynas

## Abstract

**Background:**

Migraine has a negative impact on patients’ quality of life, with the frequency of attacks being associated with greater disability and poorer health status. Frequent migraine-type headaches require prophylactic treatment, which has so far been of limited effectiveness until advent of calcitonin gene-related peptide (CGRP) monoclonal antibody.

**Materials and Methods:**

A prospective analysis was conducted of data from 41 migraine patients who experienced 4 or more monthly migraine days (MMD) longer than three months. At the beginning of the study, treatment with monoclonal antibodies against CGRP (fremanezumab 225 mg or erenumab 70 or 140 g per month) was prescribed according to the indications. The effect of the medications was evaluated after 3-month period.

**Results:**

The mean age of patients was 37.17 (±11.78) years. It was found that 17 patients (41.5%) had episodic migraine (EM) and 24 (58.5%) had chronic migraine (CM). Fremanezumab was prescribed to 26 patients (63.4%) and erenumab to 15 patients (36.6%); among the latter, 13 patients used 70 mg/month and 2 patients used 140 mg/month. Three months after treatment, CM changed to EM for 19 patients (79.2%), 27 patients (65.9%) had ≥50% reduction in the number of MMD and total migraine disability assessment (MIDAS) score was reduced by >50% in 31 patients (75.6%). Also, all areas of quality of life of patients were improved after 3 months continued treatment compared to baseline.

**Conclusions:**

For more than half the patients using fremanezumab or erenumab after 3-month period, MMD decreased by ≥50% and total MIDAS score by >50 points. All areas of quality of life were improved after prophylactic treatment of migraine.

## Abstract (it)

**Įvadas:**

Migrena neigiamai veikia pacientų gyvenimo kokybę ir galvos skausmų dažnis yra susijęs su didesnia negalia ir blogesne sveikata. Esant dažnam migreniam galvos skausmui yra reikalingas profilaktinis gydymas, kurio efektyvumas, iki atsirandant biologinei terapijai, buvo ribotas.

**Metodika:**

Perspektyviniame tyrime dalyvavo 41 migrena sergantys pacientai, kuriems pasireiškė 4 ir daugiau migreninių dienų per mėnesį (MDM) ilgiau kaip 3 mėnesius. Pacientams paskirti su kalcitonino genu susijusį baltymą ir jo receptorius veikiantys (angl. calcitonin gene-related peptide, CGRP) monokloniniai antikūnai (fremanezumabas 225 mg ar erenumabas 70 mg ar 140 mg per mėnesį). Gydymo efektyvumas įvertintas po 3 mėnesių.

**Rezultatai:**

Pacientų amžiaus vidurkis buvo 37,17 (±11,78) metų. Nustatyta, kad epizodinę migreną (EM) turėjo 17 (41,5 proc.) pacientų, o lėtinę migreną (LM) – 24 (58,5 proc.) pacientai. Fremanezumabas paskirtas 26 (63,4 proc.), o erenumabas – 15 (36,6 proc.) pacientams; atitinkamai, 13 pacientų naudojo erenumabo 70 mg per mėnesį, o 2 pacientai – 140 mg per mėnesį. Po trijų mėnesių vartojant monokloninius antikūnus, LM pasikeitė į EM 19 (79,2 proc.) pacientų, ≥50 proc. MDM sumažėjo 27 (65,9 proc.) pacientams ir 31 (75,6 proc.) pacientui bendras migrenos įtakos veiklai (angl. migraine disability assessment scale, MIDAS) balas sumažėjo >50 proc. Visose srityse gyvenimo kokybės įvertinimas pagerėjo 3 mėnesius skiriant monokloninius antikūnus.

**Išvados:**

Daugiau kaip pusei pacientų sumažėjo ≥50 proc. MDM ir >50 proc. MIDAS balai, gyvenimo kokybės įvertinimas visose srityse pagerėjo po 3 mėnesių skiriant gydymą monokloniniais antikūnais.

## Introduction

Migraine is one of the most common neurological disorders, affecting more than 1 billion people worldwide [1]. According to several studies, the overall prevalence of migraine varies between 12 and 35.5%; in Lithuania, the prevalence is estimated to be 20.4% [2–4]. Migraine affects women more frequently than men and is most prevalent in the 35-to-39-year age group [1], although the incidence is highest among those aged 25-34 years old [5]. The cost associated with migraines has been estimated at between EUR 50 and 111 billion in Europe, only 7% of which is attributed to direct medical costs [6]. Such an economic burden can be explained by the fact that this condition predominantly affects young working-age individuals, causing significant disruption to their quality of life and productivity and hindering their career development [2,7,8].

Studies show that migraine has a negative impact on patients’ quality of life, with the frequency of attacks being associated with greater disability and poorer health status [9]. According to the Eurolight project, the quality of life of migraine patients in Lithuania was found to be worse than that of people who did not experience headaches [3]. In general, studies using the Migraine Disability Assessment (MIDAS) have observed that migraine patients experience the greatest limitations in terms of time they could devote to leisure activities and housework [9]. Additionally, migraine patients tend to be less active than healthy subjects and more often report sleepiness and reduced productivity during the periods between headache attacks [10]. Moreover, having two or more comorbidities is associated with worse quality of life and greater disability [11].

The guidelines for migraine treatment indicate that the goal of prophylactic treatment is not only to reduce the frequency, intensity, and duration of attacks and the use of acute headache medication, but also to improve health-related quality of life and reduce disability, headache-related psychological problems, stress, and economic costs [12–14]. However, the proportion of migraine patients who continue to be prescribed prophylactic treatment with oral medication 12 months after the initial prescription is only 20% [15]. These medications are often discontinued due to their insufficient effectiveness, side effects, and interactions with other medications [16].

Monoclonal antibodies were the first drug specifically developed for migraine prevention, and have been confirmed as effective and safe in randomized clinical trials [17,18]. Currently, the European Medicines Agency has approved four monoclonal antibodies that target the calcitonin gene-related protein (CGRP) or its receptor. So far, only fremanezumab and erenumab are available in Lithuania. As these are new medications, many questions about their effectiveness in real clinical practice, their long-term impact on migraine control and quality of life, the optimal duration of treatment, adverse effects, the indications for changing medications if they lack efficacy, and treatment outcomes remain unanswered. There are only a few published studies in Lithuania that have evaluated the effectiveness of these medications [19,20].

### Aim

The aim was to determine the effectiveness of CGRP monoclonal antibodies and their effect on disability and quality of life in migraine patients.

## Materials and Methods

### Study participants

A prospective analysis was conducted of data from 41 migraine patients at the Outpatient Neurology Department of the Hospital of the Lithuanian University of Health Sciences Kaunas Clinics between December 2020 and June 2022. The study was approved by the Bioethics Committee of the Lithuanian University of Health Sciences (BEC-LSMU®-43) and the Regional Kaunas Bioethics Committee (2022-BE-10-0013).

## Inclusion criteria

1. Age 18 years or older.
2. Diagnosis of migraine according to the International Classification of Headache Disorders, 3rd edition [1].
3. Consent to participate in the study.

## Exclusion criteria

1. Younger than 18 years.
2. Coexisting conditions that, in the researcher’s judgment, could have a significant impact on the patient’s quality of life and daily activities.
3. Use of oral medication for migraine prophylaxis.
4. Prior treatment with monoclonal antibodies for migraine.

**Figure 1:**
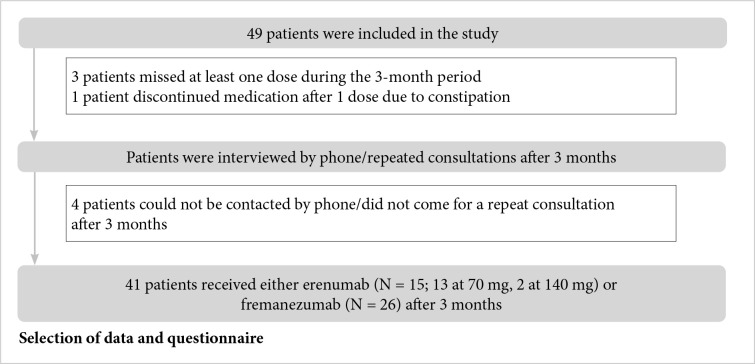
Study diagram and patient flow.

Patients who experienced 4 or more days of migraine headache were included in the study. At the beginning of the study, treatment with monoclonal antibodies against CGRP (fremanezumab 225 mg ×1 once a month or erenumab 70 or 140 mg once a month) was prescribed according to the indications. The treating neurologist decided which medication (receptor or ligand-acting monoclonal antibody) to administer based on the adverse effects. A diagram of the study and patient flow is presented in Figure 1.

## Questionnaires and data collection

Patients were asked to complete a demographic and clinical questionnaire (gender, age, pain intensity assessment (numeric pain intensity scale), medication used during headache, family history of migraine, migraine-provoking factors). Before treatment with monoclonal antibodies, patients quantified the number of migraine and other headaches, and some patients completed a written paper diary. When patients are prescribed monoclonal antibody treatment, it is recommended that they fill out a paper diary marking the days of migraine and other headaches. Additionally, data on the type of migraine (with/without aura) and prescribed medication were collected from the medical records. Anxiety, depression, and other patient-reported outcomes were not assessed in this study. Also, no data were collected on acute medication doses and which specific triptans were used.

Two questionnaires were used in this study: MIDAS and the 36-Item Short Form Health Survey (SF-36). Patients completed the questionnaires twice: before being prescribed monoclonal antibodies against CGRP and after continuing treatment for 3 months. The MIDAS questionnaire consists of 5 questions, by which patients indicate the number of days in the last 3 months they were absent from work/school due to headaches, experienced reduced work capacity or ability to study (more than half), were unable to do housework at all, had lower productivity in this activity (more than half), and had less ability to engage in social activities, communication with family members, and enjoy free time. The final MIDAS score is obtained by summing up the days with headaches indicated in all questions: 0–5 days: little or no disability (grade I); 6–10 days: mild disability (grade II); 11–20 days: moderate disability (grade III); >21 days: severe disability (grade IV) [21]. The SF-36 consists of 36 questions assessing 8 domains, evaluating physical functioning, limitations due to physical health, limitations due to emotional problems, energy/fatigue, mental health, social functioning, pain, and general health. The results are calculated according to formulas specified by the authors. The score for each domain ranges from 0 to 100 points [22].

Patients were re-interviewed after 3 months. The collected data included number of monthly migraine days (MMD) and monthly headache days (MHD), and answers to MIDAS and SF-36 questionnaires.

The effectiveness of prophylactic treatment with monoclonal antibodies was evaluated according to the following measures: number of MMD before starting prophylactic treatment and after 3 months, having ≥ 50% reduction in MMD after 3 months, number of medications used for migraine headaches before starting treatment with monoclonal antibodies and after 3 months, having >50% reduction in MIDAS score after 3 months, and health-related quality of life scores before starting treatment and after 3 months.

## Statistical analysis

The data were processed and analyzed using Microsoft Excel and SPSS 27.0. The normality of the sample distribution was assessed by the Kolmogorov–Smirnov test. Quantitative values were determined by calculating mean with standard deviation (SD) for parametric data or median with interquartile range (IQR) for nonparametric data. Qualitative values were determined as frequencies and relative frequencies (percentages) of the values of the characteristic under study. Data were analyzed using the Mann–Whitney U test, χ2 test, and Wilcoxon test. A significance level of p < 0.05 was considered statistically significant.

## Results

**Figure 2:**
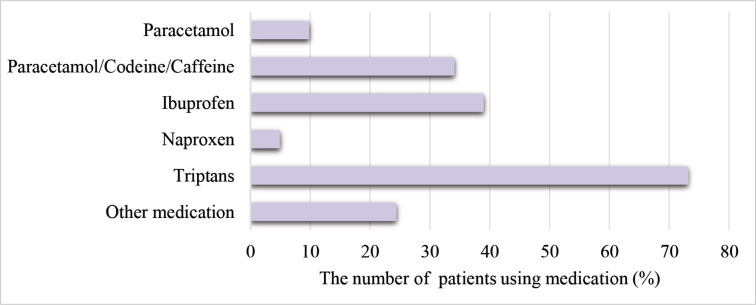
Medications used for acute migraine headache.

The mean age of patients was 37.17 (±11.78) years, and 36 women (87.8%) participated in the study. Nine patients (22%) had migraine with aura. It was found that 17 patients (41.5%) had episodic migraine (EM) and 24 (58.5%) had chronic migraine (CM), and 11 patients (26.8%) had a family history of migraine. The most frequently used medication was triptans (N = 30, 73.2%) (Figure 2).

**Figure 3:**
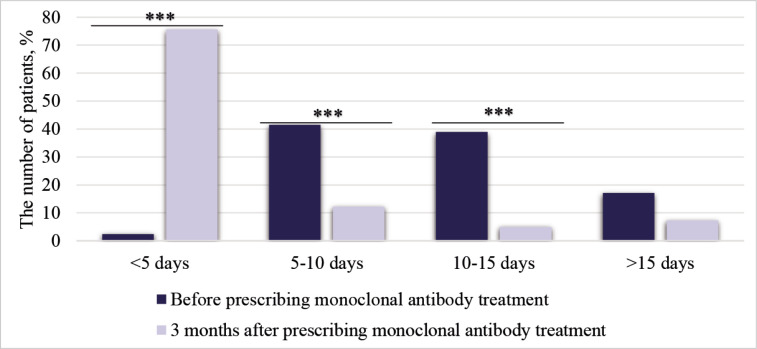
Number of days per month patients required medication for migraine headaches before and 3 months after treatment with monoclonal antibodies; ***p < 0.001.

Fremanezumab was prescribed to 26 patients (63.4%) and erenumab to 15 patients (36.6%); among the latter, 13 patients used 70 mg/month and 2 patients used 140 mg/month. Three months after treatment, 27 patients (65.9%) had ≥50% reduction in the number of MMD. Also, a statistically significant decrease in pain intensity was observed 3 months after using monoclonal antibodies (Table 1). CM changed to EM for 19 patients (79.2%), and CM remained for 5 patients (20.8%). The number of days when medication was needed to relieve migraine headaches was assessed. After 3 months of using monoclonal antibodies, most of the patients reported needing medication for headache relief <5 days/month (Figure 3).

**Figure 4:**
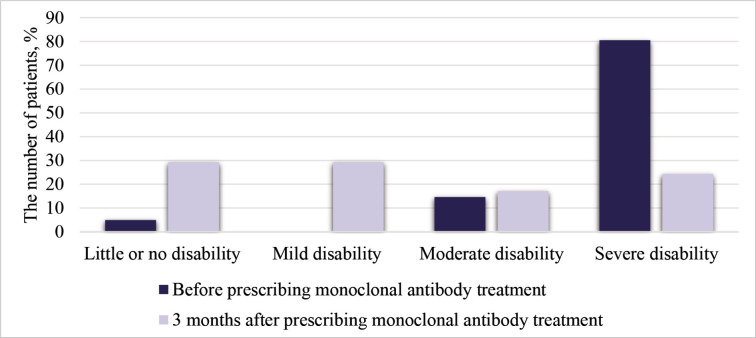
MIDAS scores in group with prophylactic treatment before and 3 months after using monoclonal antibodies; ***p < 0.001.

The median of the total MIDAS score was statistically significantly lower after 3 months (45 (IQR 25–66) and 8 (IQR 3.5–19), p < 0.001). The total MIDAS score was reduced by >50% in 31 patients (75.6%) after 3 months of treatment with monoclonal antibodies. Migraine had a significant impact on activities of daily living before treatment with monoclonal antibodies and little or no impact during treatment (p < 0.001) (Figure 4). All areas of quality of life were improved 3 months after treatment compared to baseline (p < 0.05) (Table 1).

**Table 1:** Clinical characteristics and health-related quality of life before and 3 months after prophylactic treatment with monoclonal antibodies.

	Before administration of monoclonal antibodies	3 months after administration of monoclonal antibodies	p-value
**Clinical characteristics of patients**			
Monthly migraine days (MMD), median (IQR)	10 (6–15)	4 (2–6)	<0.001
Monthly headache days (MHD), median (IQR)	5 (0–14.5)	1 (0–3.5)	<0.001
Pain intensity (numeric pain intensity scale), median (IQR)	9 (7–9)	6 (6–8)	<0.001
**SF-36**			
Physical functioning	85 (75–95)	95 (87.5–100)	<0.001
Limitations due to physical health	50 (12.5–75)	100 (62.5–100)	<0.001
Limitations due to emotional problems	66.7 (33.3–100)	100 (66.7–100)	<0.001
Energy/fatigue	55 (40–67.5)	65 (57.5–70)	<0.001
Emotional well-being	64 (52–76)	72 (64–80)	<0.001
Social functioning	62.5 (50–75)	75 (62.5–87.5)	<0.001
Pain	45 (35–56.3)	67.5 (50–80)	<0.001
General health	45 (30–60)	55 (45–70)	0.001

IQR, interquartile range; SF-36, 36-Item Short Form Health Survey.

**Figure 5:**
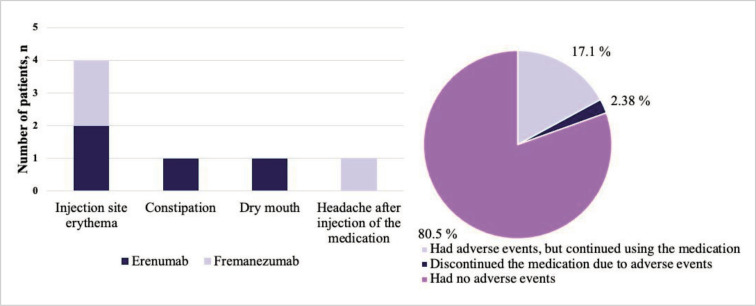
Adverse effects of erenumab and fremanezumab.

Adverse effects were experienced by 4 patients when using erenumab: constipation (1 patient), dry mouth (1 patient), and skin reaction (redness) at the injection site (2 patients) (Figure 5). Adverse effects were experienced by 3 patients when using fremanezumab: skin reaction (redness) at the injection site (2 patients) and headache after injection (1 patient). Only one patient discontinued erenumab due to constipation, the other patients did not discontinue treatment due to an adverse effect (injection site erythema, dry mouth, headache after injection of the medication) (Figure 5).

## Discussion

To the best of our knowledge, this is the first prospective study evaluating the effectiveness of monoclonal antibodies (erenumab and fremanezumab) targeting CGRP and their impact on the quality of life of migraine patients in Lithuania. Previously, only a few retrospective studies were published on this topic in Lithuania [19,20].

The regulations for reimbursement of monoclonal antibody treatment against CGRP for migraine prophylaxis differ from country to country. This leads to different uses in daily clinical practice with respect to the number of previously prescribed prophylactic medications and need for therapy breaks [23]. Monoclonal antibodies can be prescribed as first-line prophylactic treatment, and it is recommended, but not mandatory, to prescribe two oral drugs of different classes in Lithuania. The data comparison is complicated by the varying order of administration of monoclonal antibodies, evaluation of treatment efficiency (paper or electronic diary), and lack of validated questionnaires in different countries [23].

Our study included more women than men. These data coincide with systematic reviews according to real-world data [23,24]. In this study, one-fifth of the patients had auras. However, not all studies collect data on auras [23]. Our study showed that more patients were likely to use two or more types of medication and were more likely to use triptans in combination with NSAIDs. This may be related to the frequency of migraine headaches, as only 21–50% of patients are satisfied with their treatment for acute pain [25]. Additionally, inadequate treatment efficacy can lead to progression from EM to CM, which is more difficult to manage and has a greater impact on quality of life [26]. In this study, more patients had CM than EM before using monoclonal antibodies against CGRP. Other studies also included patients with EP and CM, but there are studies of patients with CM only [24]. Also, in many studies, patients have been diagnosed with medical overuse headache. However, in our study, medication overuse headache was not evaluated due to a lack of data. The results of our study show that the larger number of patients with CM using several different types of acute medication indicates a need for prophylactic migraine treatment.

In our study, patients used erenumab at either 70 or 140 mg per month or fremanezumab at 225 mg per month. These medications were prescribed based on the indications valid in Lithuania. A higher proportion of patients used fremanezumab 225 mg per month than erenumab 70 mg per month. In a systematic review of real-world data by Pavelic et al., erenumab was prescribed in more studies, while erenumab or fremanezumab were prescribed in fewer studies [23]. In another systematic review, a larger proportion of studies included more patients prescribed erenumab 70 mg or 140 mg per month [24]. In our study, regarding the effect of monoclonal antibodies after 3 months, it was observed that patients experienced fewer migraine days and other headache days per month. Notably, 65.9% of subjects achieved a ≥50% reduction in MMD and 58.5% achieved a ≥50% reduction in MHD. This aligns with the findings in a review by Pavelic et al., which showed that, on average, 44% of patients achieved a ≥50% reduction in MMD after 3 months, and this increased with longer use of the medication. Similar effectiveness was found in an overall assessment showing a ≥50% reduction in MHD [23]. However, more patients, especially those with CM, achieved a >50% reduction in MIDAS scores [27]. In this study, more subjects achieved a >50% reduction in MIDAS scores (75.6%) than a reduction in MMD (65.9%). Although this questionnaire is not often used in clinical practice in Lithuania due to time constraints, it could be used as an additional method to evaluate treatment effectiveness. At the moment, the lack of a standardized questionnaire for assessing quality of life in migraine patients poses challenges in research, as various questionnaires are used. Unfortunately, in Lithuania we still have only one validated questionnaire for assessing general health-related quality of life (SF-36). A standardized questionnaires could allow better comparison of results, thus enhancing the quality of evidence in the future. Despite these challenges, both our study and studies reported in the literature show improved quality of life after 3 months of monoclonal antibodies [28–30].

In this study, 17.1% subjects experienced side effects, the most common being a local skin reaction after injection (redness, pain) with both erenumab and fremanezumab. A systematic review of real-world data also found that redness of the skin after injection is the second most common side effect, and constipation is the first [23]. The results of our study may have been influenced by the small size of the group treated with erenumab, as its use has been associated with constipation. In this study, only one subject discontinued erenumab after 1 dose due to constipation. The rate varies in the literature, but on average, about 5.9% of patients discontinue the medication, which a much lower rate compared to other migraine prevention medication [15,23].

This study had several limitations. Previously used prophylactic migraine treatment and disease duration were not included in the study data, because the subjects were not always able to name which medication they used and how long they had migraines. This was also not recorded in the medical documentation. Treatment efficacy at 3 months was not compared between EM and CM groups due to the small size of the study sample. Also, the efficacy of erenumab and fremanezumab was not compared due to the small number of patients. The comparison of the data with the results of other studies is limited due to the lack of validated quality of life questionnaires in Lithuania, and there is currently no standardized methodology for assessing the quality of life of migraine patients.

## Conclusions

For more than half the patients using fremanezumab or erenumab after 3-month period, MMD decreased by ≥50% and total MIDAS score by >50 points. Also, more than half of the patients required acute headache medication <5 days/month after 3 months of treatment with monoclonal antibodies. All areas of quality of life were improved after prophylactic treatment. Three months after using monoclonal antibodies, 17.1% of patients had an adverse event, the most common of which a local dermal allergic reaction. One patient discontinued erenumab after 1 dose due to constipation.
